# Patent Foramen Ovale and Pregnancy: A Case Report and Literature Review

**DOI:** 10.7759/cureus.84973

**Published:** 2025-05-28

**Authors:** Diego González Guzmán, Carlos A Andrade-Castellanos, Marco Antonio Ponce-Gallegos, Alejandra García Valencia

**Affiliations:** 1 Internal Medicine, Hospital Civil de Guadalajara Dr. Juan I. Menchaca, Guadalajara, MEX; 2 Cardiology, Instituto Nacional de Cardiología Ignacio Chávez, Mexico City, MEX; 3 Internal Medicine, Health Science Center, Universidad de Guadalajara, Guadalajara, MEX

**Keywords:** atrial fibrillation, hypercoagulable state, patent foramen ovale (pfo), postpartum period, stroke

## Abstract

Patent foramen ovale (PFO) is a common congenital cardiac anomaly that can serve as a conduit for paradoxical embolism, particularly in young patients with cryptogenic stroke. Pregnancy induces a hypercoagulable state that may unmask underlying predisposing conditions such as PFO. Although ischemic stroke during pregnancy is rare, it carries significant maternal and neonatal complications.

We report the case of a young woman in her third trimester who presented with an acute ischemic stroke. Initial diagnostic evaluation using transthoracic echocardiography (TTE) with a bubble study confirmed the presence of a PFO, characterized by a large right-to-left shunt. The Risk of Paradoxical Embolism (RoPE) score was high, suggesting a strong likelihood that the PFO was causally related to the stroke. No alternative etiology was identified after thorough investigation.

She was managed with anticoagulation therapy, with input from a multidisciplinary team including neurology, cardiology, and maternal-fetal medicine specialists. Postpartum, she underwent successful percutaneous PFO closure with a Gore Cardioform device (W. L. Gore & Associates, Inc., Newark, DE), with follow-up imaging revealing minimal residual shunting and favorable neurological recovery.

This case highlights the importance of considering PFO as a potential cause of stroke during pregnancy. It underscores the need for individualized management guided by anatomical characteristics and validated clinical scoring systems such as the RoPE score. In contrast to some prior reports advocating early closure, this case supports deferring intervention until postpartum in selected patients, aligning with current guidelines that recommend a cautious approach during pregnancy.

## Introduction

A patent foramen ovale (PFO) is a congenital cardiac anomaly resulting from the incomplete closure of the interatrial septum and is the most prevalent cause of right-to-left circulatory shunting in adults. The pathophysiological mechanism by which a PFO contributes to stroke is paradoxical embolism, whereby a venous thromboembolism bypasses the pulmonary circulation via a right-to-left shunt and enters the systemic arterial system, potentially occluding cerebral vessels. PFO has also been associated with other conditions such as decompression sickness, secondary migraine, and platypnea-orthodeoxia [1].

Cardioembolic stroke accounts for about 30% of all ischemic strokes and is associated with higher morbidity and mortality [2]. While high-risk sources such as atrial fibrillation (AF), infective endocarditis, and left ventricular thrombus are well-recognized, lower-risk sources such as PFO may still be clinically significant, especially in younger patients with no other apparent cause [3].

Stroke during pregnancy is rare, with an estimated incidence of 30 per 100,000 deliveries, but it is a leading cause of severe maternal morbidity and long-term disability postpartum [4]. Risk factors include hypertensive disorders of pregnancy (e.g., preeclampsia), thrombophilias, and peripartum cardiomyopathy [5]. In this context, the physiological hypercoagulable state of pregnancy may unmask a previously silent PFO, increasing the Risk of Paradoxical Embolism (RoPE) in susceptible individuals. The hypercoagulable state of pregnancy, driven by increased coagulation factors and decreased fibrinolysis, augments the risk of venous thromboembolism. In the presence of a PFO, this physiological change increases the likelihood of paradoxical embolism, particularly during periods of increased right atrial pressure, such as labor, Valsalva maneuvers, or postpartum recovery [2,5].

Although early clinical trials did not demonstrate a clear advantage of interventional closure over medical therapy, more recent randomized controlled trials, including the Randomized Evaluation of Recurrent Stroke Comparing PFO Closure to Established Current Standard of Care Treatment (RESPECT), Patent Foramen Ovale Closure or Anticoagulants versus Antiplatelet Therapy to Prevent Stroke Recurrence (CLOSE), and GORE® HELEX® Septal Occluder/GORE® CARDIOFORM Septal Occluder and Antiplatelet Medical Management for Reduction of Recurrent Stroke or Imaging-Confirmed TIA in Patients With PFO (REDUCE), and subsequent meta-analyses have shown a significant reduction in recurrent stroke rates with percutaneous PFO closure in selected patients with cryptogenic stroke and high-risk PFO features [6,7]. These studies have shifted clinical guidelines toward favoring closure in well-selected patients while emphasizing individualized risk assessment.

## Case presentation

**Figure 1:**
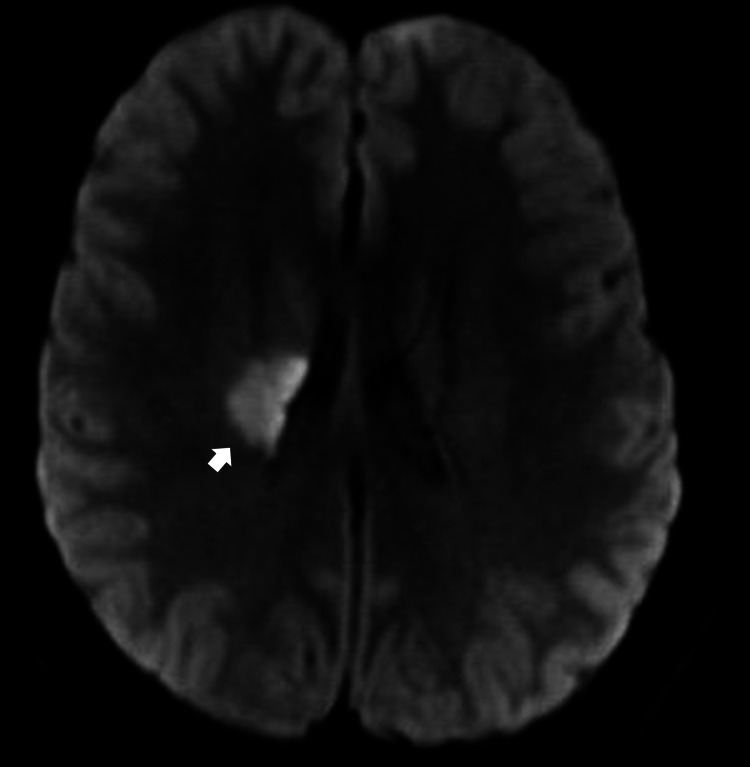
Axial slice in T FLAIR sequence shows a hyperintense area located in the tail of the right putamen and the posterior limb of the ipsilateral internal capsule and extending toward the corona radiata. FLAIR: fluid-attenuated inversion recovery

**Figure 2:**
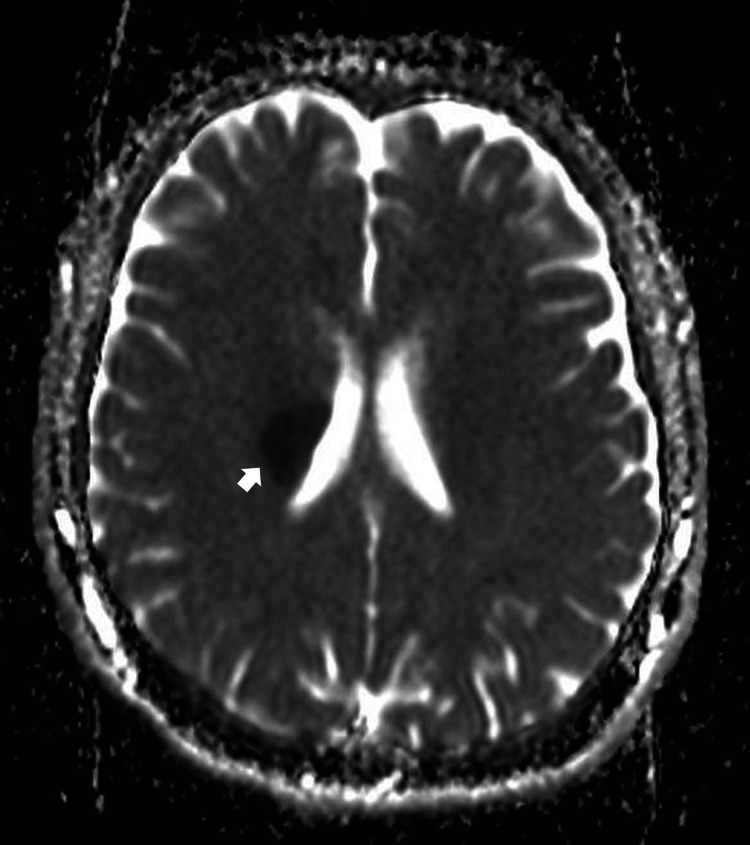
Axial slice in ADC sequence with signal restriction, corresponding to an area of acute ischemia. ADC: apparent diffusion coefficient

**Figure 3:**
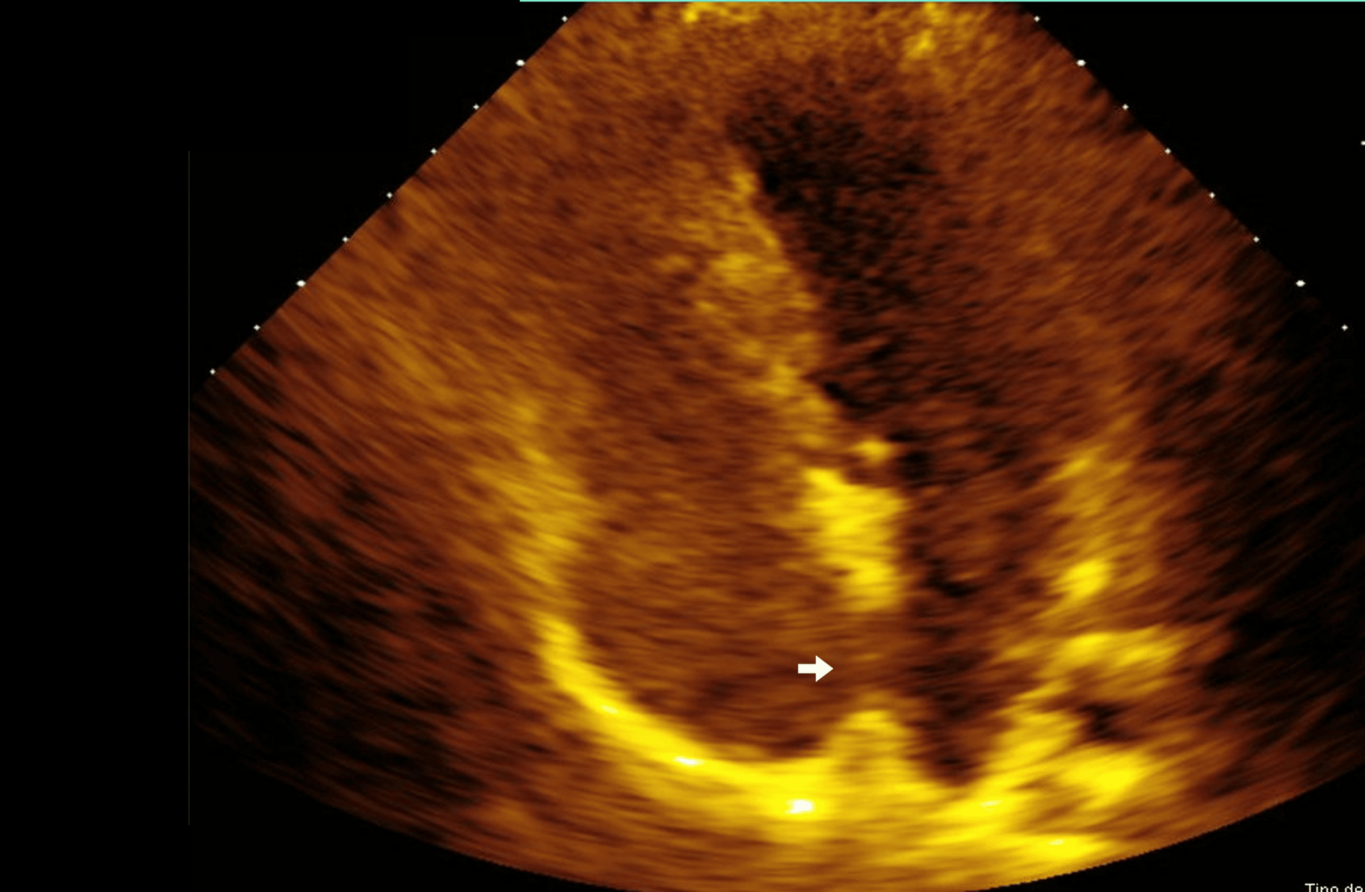
Transthoracic echocardiogram in a four-chamber apical view, showing a grade II patent foramen ovale (passage of 5-20 bubbles within the first five heartbeats).

A 26-year-old right-handed woman, 27 weeks pregnant, presented with acute left-arm hemiplegia and left-leg hemiparesis. The patient was watching television around 6:00 p.m. when she experienced sudden-onset weakness on the left side of her body. While attempting to stand up to use the bathroom, she collapsed due to impaired postural support from left-sided hemiparesis. There were no preceding symptoms such as headache, nausea, or visual changes. Upon arrival at the emergency room, she was outside the thrombolysis window and had a National Institutes of Health Stroke Scale (NIHSS) score of 10. Neurological examination revealed left central facial palsy, mild dysarthria (slurred but comprehensible speech), preserved sensation, flaccid paralysis of the left arm (0/5), and moderate weakness in the left leg (2/5) on the Daniels scale. Magnetic resonance imaging (MRI) showed an acute ischemic focus in the right periventricular white matter, the posterior limb of the internal capsule, and the caudal margin of the ipsilateral putamen, with no signs of hemorrhagic transformation (Figures 1, 2). These findings are consistent with the disruption of descending corticospinal tract fibers within the internal capsule, explaining the contralateral motor deficits. Carotid Doppler ultrasound showed laminar flow without atheromatous plaques. She had a low-density lipoprotein level of 97 mg/dL. A comprehensive thrombophilia screen, including factor V Leiden, protein C and S, antithrombin III, lupus anticoagulant, anticardiolipin, and anti-β2 glycoprotein I antibodies, was performed during pregnancy. Although results were within normal limits, the interpretation warrants caution, as physiological changes in pregnancy (e.g., increased fibrinogen and reduced protein S activity) can affect test reliability. A 24-hour Holter monitor revealed no arrhythmias. Given the patient’s young age, absence of structural heart disease, and low clinical suspicion for atrial fibrillation, prolonged cardiac monitoring was not deemed necessary. Transthoracic echocardiography (TTE) revealed a grade II PFO (Figure 3). Doppler ultrasound of the lower extremities was also performed to evaluate for deep vein thrombosis as a potential embolic source, and no evidence of thrombus formation was found.

Initial management included therapeutic enoxaparin throughout the remainder of the pregnancy, combined with physical rehabilitation and close monitoring. This strategy was guided by a multidisciplinary team including neurology, cardiology, and maternal-fetal medicine, considering the hypercoagulable state of pregnancy and the presence of a high-risk PFO. The patient showed gradual improvement in her left arm strength and was discharged with follow-up planning. She underwent an elective cesarean section at 37.5 weeks to minimize the risks associated with her condition. After pregnancy, a transesophageal echocardiogram (TEE) revealed a grade III PFO and a windsock-shaped left atrial appendage with type 1 flow, free of thrombi, as well as a slightly lax but intact non-aneurysmal interatrial septum. The windsock-shaped morphology and type 1 flow pattern are associated with streamlined laminar flow and low thromboembolic risk.

**Figure 4:**
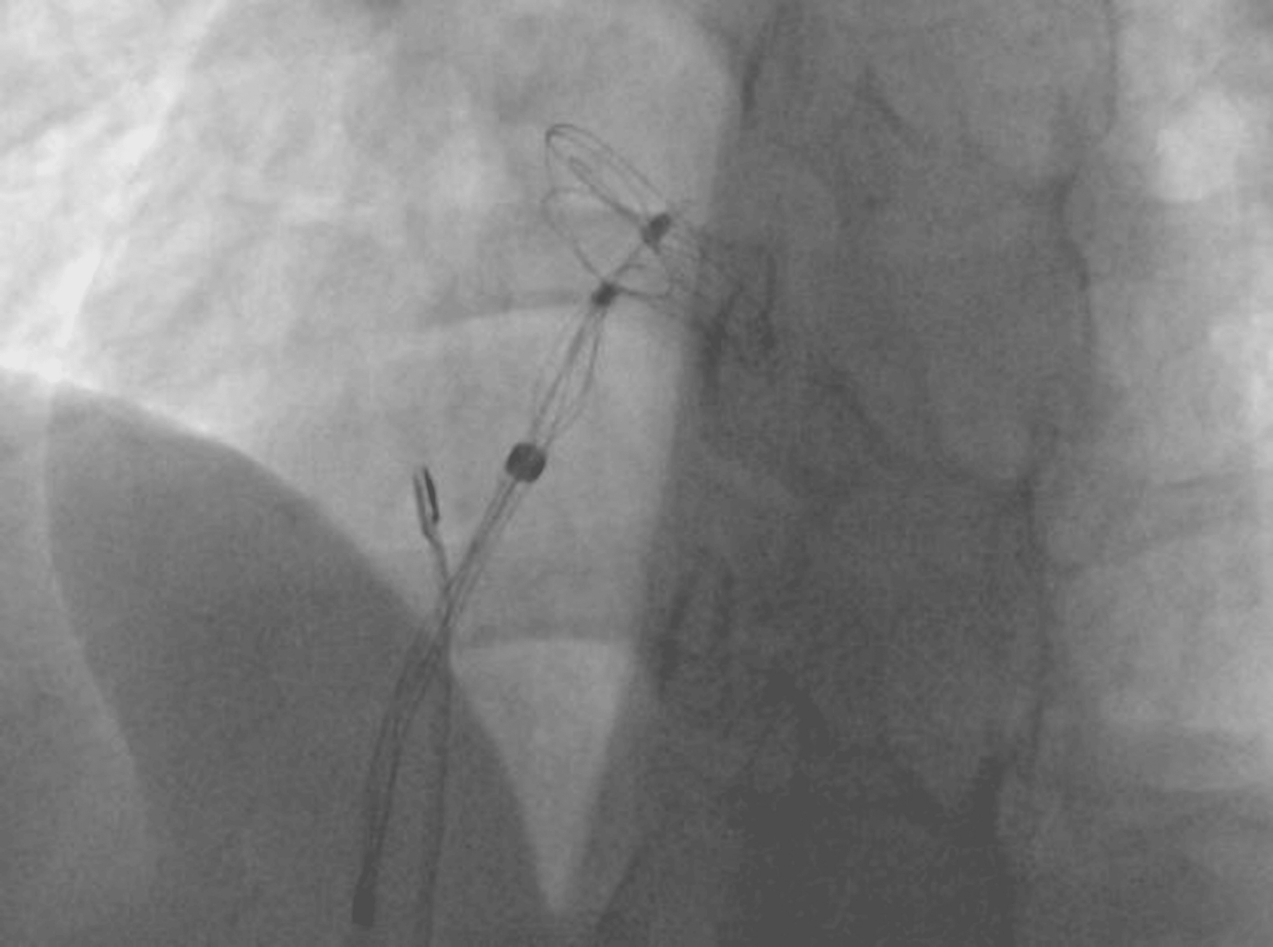
Catheterization showing the placement of the Gore device introducer at the level of the left atrium.

**Figure 5:**
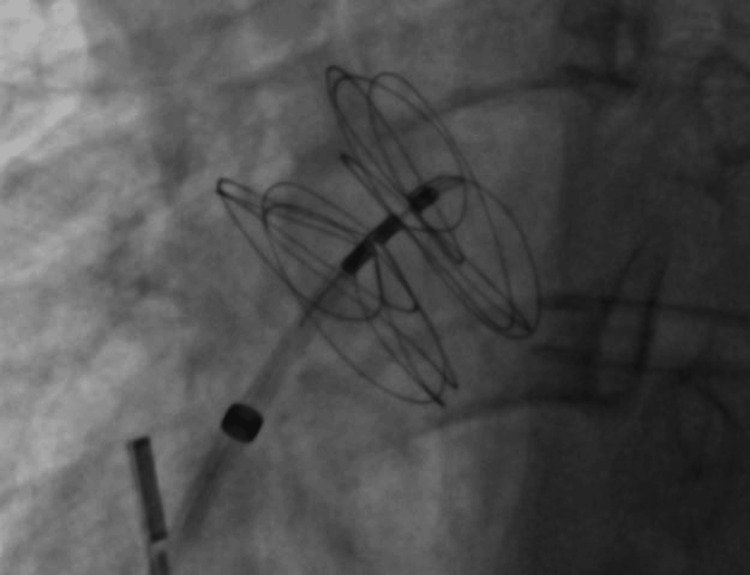
Catheterization showing the Gore device implanted in the interatrial septum.

**Figure 6:**
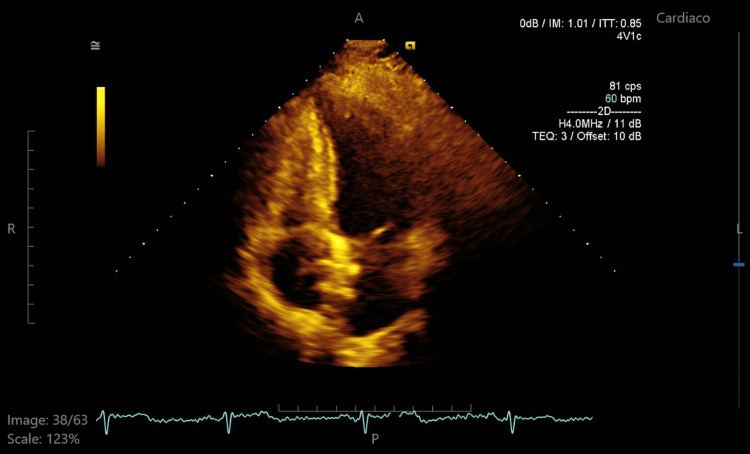
Four-chamber apical view in transthoracic echocardiography showing the Gore device in the interatrial septum.

**Figure 7:**
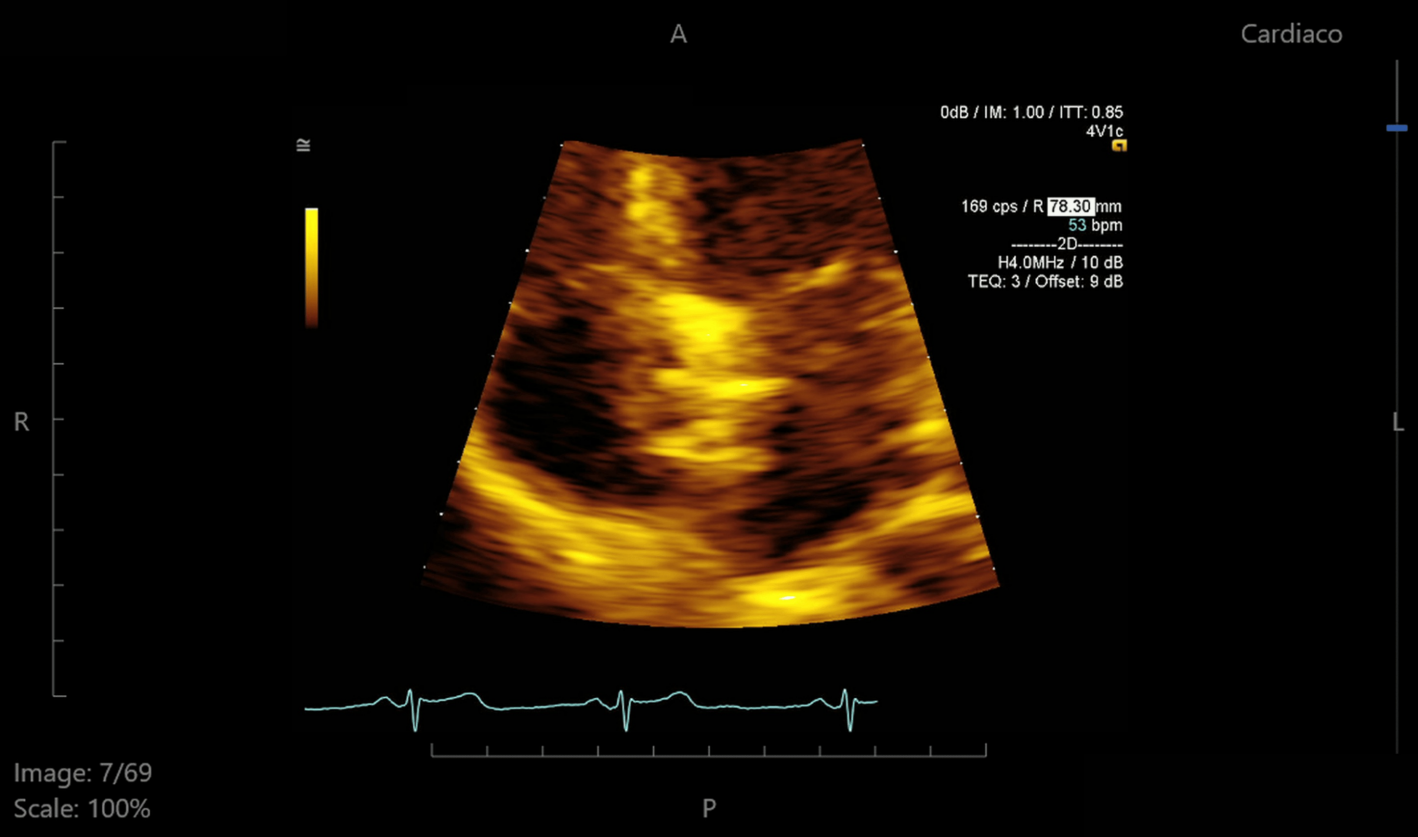
Zoomed apical four-chamber view highlighting the Gore Cardioform device at the interatrial septum, confirming its position and stability.

**Figure 8:**
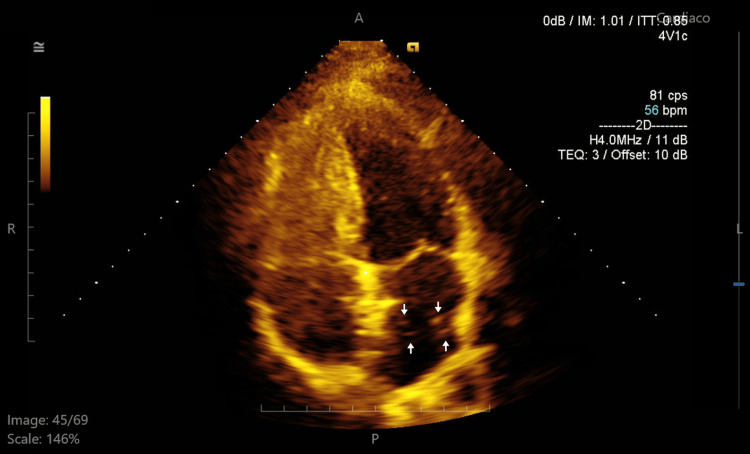
Four-chamber apical view in transthoracic echocardiography showing the passage of a few bubbles through the distal edge of the device.

In our patient, pregnancy and a PFO were significant risk factors for stroke. She had a RoPE score of 9, indicating an 84% probability that the stroke was attributable to the PFO, and also presented with a large interatrial shunt, further increasing her embolic risk. Given these factors and her desire for future pregnancies, she underwent cardiac catheterization, during which a Gore Cardioform device (W. L. Gore & Associates, Inc., Newark, DE) was advanced through the PFO into the left pulmonary vein and deployed at the interatrial septum (Figures 4, 5). The procedure was completed without complications, and the Minnesota maneuver was performed to assess proper anchoring. It involves gentle traction on the device prior to its release, confirming stability and the absence of residual shunt. She was treated with clopidogrel for three months following closure and then continued on aspirin for long-term secondary prevention. Statins were also initiated postpartum. Although not strictly indicated in the absence of atherosclerosis or dyslipidemia, its use was considered on a case-by-case basis, taking into account potential vascular and anti-inflammatory benefits, as well as institutional practice. At follow-up, transthoracic echocardiography (Figures 6-8) showed minimal residual bubble passage and no arrhythmias. Her neurological recovery was excellent, with a muscle strength of 4/5 in her left arm and 5/5 in her left leg, a modified Rankin Scale (mRS) score of 1, and a Barthel Index of 100, indicating complete independence in activities of daily living. She also received counseling for future pregnancies, including the need for close monitoring and the continuation of antiplatelet therapy under specialist guidance.

## Discussion

Hypercoagulable state and stroke in pregnancy

Pregnancy-induced hypercoagulability reduces the risk of bleeding during childbirth and the postpartum period but significantly increases the risk of thromboembolic events. Pregnant women have a 4-5-fold higher risk of thromboembolism compared to nonpregnant women, with 80% of cases being venous in origin [8]. This prothrombotic state is driven by elevated levels of coagulation factors (VII, VIII, X, and von Willebrand factor), increased fibrinogen and plasminogen activator inhibitor type 1, decreased fibrinolysis, and a reduction in natural anticoagulants such as protein S. Additionally, resistance to activated protein C rises. These changes begin early in pregnancy and peak during the third trimester, with the risk of venous thromboembolism rising 20-80-fold during the first six postpartum weeks [8-10].

PFO in cryptogenic stroke

The foramen ovale, a fetal structure enabling right-to-left shunting, usually closes functionally at birth and anatomically by around seven months of age [11]. However, incomplete closure results in a PFO, a congenital anomaly found in approximately 20%-25% of the adult population [12]. Among individuals with cryptogenic stroke, 40%-50% are found to have a PFO [13]. The pathogenic significance of a PFO varies depending on anatomical features. Patients with large shunts (more than 20 microbubbles in the left atrium within three cardiac cycles after right atrial opacification) or an atrial septal aneurysm (ASA), defined as septum primum excursion of ≥10 mm from the atrial septal plane, are at higher risk for recurrent stroke and derive greater benefit from PFO closure. Other associated features, such as a prominent Eustachian valve or Chiari network, may further increase the Risk of Paradoxical Embolism [12-15]. The interaction between the hypercoagulable state of pregnancy and an anatomically high-risk PFO increases the likelihood of paradoxical embolism. In this context, tools such as the RoPE score help determine causal association and support closure decisions.

Although multiple diagnostic techniques are available for the detection of a PFO, TEE combined with agitated saline contrast continues to be considered the reference standard, particularly in stroke patients. It is a semi-invasive method that provides direct visualization of the PFO anatomical characteristics (size, location, and length of the tunnel); allows differentiation between a PFO and an atrial septal defect, the presence of ASA, hypermobility, the thickness of the septum primum and secundum, and the presence/absence of a Eustachian valve or Chiari network; and can identify other potential sources of embolism, such as left atrial appendage thrombus and left ventricular thrombus [12-14]. The brachial vein has traditionally been the preferred site for contrast injection to detect PFO. However, research has shown that using the femoral vein increases sensitivity in identifying a PFO. This difference is mainly attributed to the direction of venous blood flow into the right atrium. When contrast is injected through the brachial vein, it enters the right atrium via the superior vena cava and typically flows directly toward the tricuspid valve. In contrast, when injected through the femoral vein, the contrast enters via the inferior vena cava, directing it toward the superior aspect of the interatrial septum. This flow pattern, especially in the presence of a Eustachian valve, enhances the likelihood of contrast crossing a PFO if it is present [15].

For managing PFO, the PFO-Associated Stroke Causal Likelihood (PASCAL) risk stratification tool is useful for categorizing patients into three subgroups: unlikely, possible, and probable stroke-related PFO. This tool assesses two domains: the presence of high-risk features (such as a large shunt or ASA) and the RoPE score (which identifies PFOs likely related to cryptogenic stroke). Patients with a RoPE score greater than 7 and high-risk features (in the possible or probable subgroups) benefit more from PFO closure, while those in the unlikely subgroup are better suited to medical management [12,13]. The patient had a RoPE score of 9 and a large PFO, both of which place her in the “probable” category for PFO-related stroke in the PASCAL classification, supporting closure. While the role of anticoagulation in patients with cryptogenic stroke and PFO remains debated, in this case, therapeutic enoxaparin was initiated due to the high-risk anatomical features of the PFO, a high RoPE score, and the prothrombotic state of pregnancy. The decision was made in coordination with a multidisciplinary team, prioritizing maternal and fetal safety.

The selection of device and management strategy should be informed by the evidence from clinical trials [16-19]. Among the major randomized controlled trials evaluating PFO closure, the REDUCE and DEFENSE-PFO trials are most relevant to the present case due to their focus on high-risk PFO features and the devices employed. The REDUCE trial evaluated the Gore Helex and Cardioform devices in 664 patients with recent cryptogenic stroke and confirmed PFO. Over a median follow-up of 3.2 years, ischemic stroke occurred in 1.4% of patients in the closure group versus 5.4% in the medical therapy group (p = 0.002) [20]. The DEFENSE-PFO examined the Amplatzer device in 120 patients with high-risk PFO characteristics, such as large shunts or ASA. During the two-year follow-up, the composite primary outcome, which included stroke, vascular death, and major bleeding, was observed exclusively in the medical therapy group (12.9% versus 0%; p = 0.013). Ischemic stroke was also significantly lower in the closure group (10.5% versus 0%; p = 0.023) [21]. A meta-analysis of these trials indicated that device closure reduced the annualized incidence of stroke compared to medical therapy alone (0.47% versus 1.09%; adjusted HR, 0.41; p < 0.001). Risk reduction varied by PFO-related stroke likelihood: hazard ratios were 1.14 for unlikely PFO-related strokes (not significant), 0.38 for possibly related strokes, and 0.10 for probable cases, all statistically significant [22].

The Gore Cardioform device was specifically chosen in this case due to its proven efficacy in the REDUCE trial, favorable safety profile, and compatibility with atrial anatomy often seen in women of childbearing age. Its soft, conformable structure may reduce the risk of erosion and perforation, which is particularly relevant when considering future pregnancies and the hemodynamic changes they entail. Other factors influencing device selection include the size and flexibility of the closure mechanism, operator familiarity, institutional availability, and patient-specific anatomical considerations such as tunnel length or septal thickness [20].

In pregnant patients, the timing of closure requires careful consideration due to the physiological hypercoagulability and the increased risk of thromboembolic events. Although pregnancy itself is not an absolute contraindication to closure, performing the procedure postpartum may offer a safer window in most cases [23].

Individualized risk-benefit analysis is essential and should incorporate clinical features, imaging findings, patient preferences, and reproductive planning. Institutional protocols must promote the collaboration of multidisciplinary teams, including specialists in obstetrics, neurology, and cardiology, to optimize timing, safety, and long-term outcomes. Such collaboration is particularly important in female patients of reproductive age with a history of cryptogenic stroke, where decisions regarding antithrombotic therapy and PFO closure have broader implications. After PFO closure, long-term follow-up is vital to monitor for complications such as AF, which occurs in approximately 5% of patients and is often transient, especially within the first 45 days [24]. Current recommendations support the use of dual antiplatelet therapy (typically aspirin and clopidogrel) for the first 1-5 months post-closure, followed by lifelong single antiplatelet therapy in most cases [24]. Counseling regarding future pregnancies should include a review of hypercoagulability risks, the need for thromboprophylaxis during gestation and postpartum, and coordination with high-risk obstetric services.

## Conclusions

This case highlights the complex clinical decision-making required when managing cryptogenic stroke during pregnancy, particularly in the setting of a high-risk PFO. The use of structured tools such as the RoPE score and the PASCAL classification proved critical in establishing the likely causal relationship between the PFO and the stroke, thereby guiding the multidisciplinary team toward a tailored management plan. This case underscores the importance of rapid stroke evaluation in pregnancy, balancing maternal safety with fetal considerations and the need for effective secondary prevention. While intravenous thrombolysis with alteplase remains a therapeutic option in selected pregnant patients, it was not pursued in this case due to presentation outside the therapeutic window.

A key aspect of this case is the timing of PFO closure, which was intentionally deferred until the postpartum period to avoid procedural risks during pregnancy and to better align with the patient’s plans for future pregnancies. This approach exemplifies how reproductive planning must be integrated into stroke care for women of childbearing age. The patient’s risk of post-procedural AF was proactively addressed through close cardiologic follow-up, given the recognized early post-closure AF risk. Fortunately, no arrhythmias occurred, and the patient has remained neurologically intact, with no recurrence of stroke symptoms or other complications during follow-up. Her recovery reinforces the value of individualized, guideline-informed management strategies in optimizing outcomes for women experiencing stroke during pregnancy.
